# 

**Published:** 1888-04

**Authors:** 


### EDITORIAL.

### The American Medical Association.

The time for the annual meeting of this body draws nigh. The indications are that it will be largely attended, and perhaps more than ever before. This meeting promises to be a profitable and interesting one. The medical profession seems from year to year to be taking more interest in this, the leading medical association of this country. A dental section was organized in this association some eight or ten years ago. The number in attendance upon this section has hitherto of course been limited from the fact that only those having the M. D. were admitted. At the meeting of last year, held in Chicago, by a unanimous resolution of the body, this was changed so that any dental graduate who has been properly educated in the fundamental branches of medical science may become a member of the body upon the same footing and in the same way as any regular medical graduate. This, of course, largely increases the interest of the dental profession in this association. It not only gives an important privilege, but it increases the sphere of professional activity and adds much to the responsibility of the dentist.

The indications are that there will be a good attendance upon this section. On another page of this number is given a list of the subjects upon which papers will be read and discussions had in the section.

Quite extended preparations have been made for the accommodation of the members of the association and for the social enjoyment as well. Reduced railroad and hotel rates have been secured, the particulars for which have been more definitely made known through the medical journals. It may be proper to say here, however, that the following hotels will give entertainment as specified, viz : Gibson House, Walnut Street between 4th and 5th Streets, Burnet House, on the corner of Vine and 3d Streets, and Grand Hotel, 4th and Central Ave., each at from $3 to $5 per day. The Palace Hotel, on the corner of 6th and Vine

Streets, St. James Hotel, Dennison House and Walnut Street House, from $2 to $2.50 per day. Hunt's Hotel, on Vine Street between 4th and 5th Streets, at $1.75 per day. Rooms on the European plan, fifty cents per day. In addition to this a number of first-class private boarding houses will be available at from $1.50 to $2.50 per day. All the houses here named are on lines of street railroads running direct to Music Hall, where the meetings of the association are to be held. The committee on railroads have secured a reduction on the certificate plan from all railroads entering the city of Cincinnati. The rate will be full fare coming to the meeting and one-third fare returning. Persons coming to the Association will please ask for a blank certificate at the railroad office at the time they purchase their tickets when starting from home, which certificate will entitle the holder to a one-third fare returning. Drs. George Purviance and William Judkins are the local sub-committee on railroad and hotel accommodations.

### American Medical Association—A Partial Programme of the Section of Dental and Oral Surgery.

The following papers will be presented to be read and discussed in this Section:

Address by the Chairman.

"Etiology of Irregularities of the teeth," by E. S. Talbot, M. D., D.D.S., Chicago, Ill.

"Dentogeny," by Dr. W. C. Brittan, D.D.S., of Detroit, Mich. Discussion of this paper to be opened by Dr. M. H. Fletcher, M.D., D.D.S., of Cincinnati, Ohio.

"Heredity," by Dr. A. O. Rawls, D.D.S., of Lexington, Ky.

"Irregularities of the Teeth," by Dr. Geo. W. Keely, of Oxford, Ohio.

"Management of Children's Teeth," by E. G. Betty, D.D.S., Cincinnati, O.

A paper, subject not given, will be read by Prof. J. S. Marshall, M. D., D.D.S., Chicago, Ill.

Other papers are promised, but subjects not given. It is desirable that all who propose to present papers upon this occasion notify the secretary, E. S. Talbot, 125 State Street, Chicago, Ill., at the earliest possible moment.

J. Taft,
Chairman of Section.

### Dental College of the University of Michigan.

EXTENSION OF REQUIREMENTS.

By recent action of the Board of Regents of the University of Michigan, the time of attendance for graduation in the Dental Department has been extended to three terms of nine months each.

Heretofore nearly one half of those who graduated, took three terms; this however was not a requirement, except for those who had no previous study of dentistry. The general approval of the three term requirement, together with the certainty of accomplishing far better work for the student, seems to give full warrent for this step. The trend of the times, seems to indicate that some progress should be made.

### The Next Meeting of the Association of American Medical Editors.

The following programme has been arranged for the meeting at Cincinnati, Monday evening preceding the meeting of the American Medical Association, May, 1888:

Meeting called at 8 P. M.

Reading of minutes.

President's address, William Porter, M.D., of St. Louis.

Report of Committee on Organization, Dr. McMurtry, Chairman, Danville, Ky.

Election of officers for ensuing year.

Questions for consideration:

1. Is the multiplicity of medical journals an advantage to the profession? To be discussed by Drs. Crothers, Hartford; Sim, Memphis; Wile, Conn.; Love, St. Louis; Culbertson, Cincinnati; Cushing, Boston; Coomes, Louisville; and Gray, Chicago.

2. How far do medical journals distributed by drug houses and manufacturers interfere with regular medical journalism? To be discussed by Drs. Reynolds, Louisville; Davis, Chicago; Shoemaker, Philadelphia; Bond, St. Louis; Connor, Detroit; Kiernan, Chicago; Thacker, Cincinnati; and Fulton, St. Paul.

Members are requested to limit their remarks to fifteen minutes, and if possible to ten. The place of meeting will be posted in all the hotels by the local committee. Arrangements can be made at this meeting for a "press dinner" for another evening during the week, but it will be impossible to conclude the business of the Association and have the dinner the same evening.

### CONTENTS.

COMMUNICATIONS.

A MONTHLY MAGAZINE DEVOTED TO THE INTERESTS OF THE

### DENTAL PROFESSION.

*Terms Reduced to* $2.00 *Per Year. Single Copies,* 25 *Cents.*

### EDITED BY PROF. J. TAFT, D.D.S.

—AND—

### Published by SAM'L A. CROCKER & CO., Ohio Dental and Surgical Depot.

The volume commences in January and closes in December.

The REGISTER being issued monthly is always fresh, therefore Indispensable to the practitioner who desires to keep posted up with the new inventions and improvements which are daily developed. Neither expense nor effort will be spared to give value and variety to its contents. Articles will be illustrated with well executed engravings whenever they will add to the interest or assist to explanation.

Its extensive circulation and frequency of issue make it one of the BEST DENTAL ADVERTISING MEDIUMS in the United States.

All subscriptions must begin with January or July.

Local or special notices, five lines or less, will be inserted for $3 each insertion; over five lines, 50 cts. per line.

All advertising bills payable in advance, or at furthest, after the issue of the first number containing the advertisement. All transient advertisements to be paid for in advance.

☞CONTRIBUTIONS SOLICITED.

Exclusively Editorial Communications should be addressed to the Editor.

The latest number of the REGISTER may be ordered with or without other goods at any time, and all letters should be addressed to

SAM'L A. CROCKER & CO., Ohio Dental and Surgical Depot, Cincinnati, O.

